# Laparoscopic appendectomy: quality care and cost-effectiveness for today’s economy

**DOI:** 10.1186/1749-7922-8-45

**Published:** 2013-11-01

**Authors:** David Costa-Navarro, Montiel Jiménez-Fuertes, Azahara Illán-Riquelme

**Affiliations:** 1 Department of Surgery, Marina Baixa Medical Center, 7 Alcalde Jaume Botella Mayor street, Villajoyosa, Alicante, Spain

**Keywords:** Laparoscopy, Acute appendicitis, Cost- effectiveness, Laparoscopic appendectomy

## Abstract

**Background:**

Open appendectomy (OA) has traditionally been the treatment for acute appendicitis (AA). Beneficial effects of laparoscopic appendectomy (LA) for the treatment of AA are still controversial.

**Aim:**

To present our technique for LA and to determine whether LA should be the technique of choice of any case of AA instead of OA.

**Material and methods:**

All cases operated for AA (February 2011 through February 2012) by means of LA or OA were prospectively evaluated. Data regarding length of stay, complications, emergency department consultation after discharge or readmission were collected. Patients were classified into four groups depending on the severity of the appendicitis. Economic data were obtained based on the cost of the disposable material. Cost of hospital stay was calculated based on the Ley de Tasas of the Generalitat Valenciana according to the DRG and the length of stay.

**Results:**

One hundred and forty-two cases were included. Ninety-nine patients underwent OA and 43 LA. Average length of stay for LA group was 2,6 days and 3,8 for OA. Average cost of the stay for OA was 1.799 euros and 1.081 euros for LA. Global morbidity rate was 16%, 5% for LA and 20% for OA.

**Conclusions:**

LA is nowadays the technique of choice for the treatment of AA.

## Introduction

Acute appendicitis (AA) is the most frequent cause of acute abdominal pain in western countries, marked with an incidence of 100/100.000 cases per year [1] and the risk of having AA is around 8% [1-3] in a lifetime. Open appendectomy (OA) has been the standard surgical procedure for the treatment of AA for over a century, since it was described by McBurney in 1894 [4] and still remains the procedure of choice in many centers [1-3]. Subsequently, due to the development of endoscopic surgery, Semm introduced the laparoscopic appendectomy (LA) in 1981 [2], rendering a minimally invasive procedure for the skin and abdomen [2,5]; although many studies published in the very early years of the 21^st^ century, comparing OA and LA, didn’t really determine a superiority of the laparoscopic approach [6-9], some more recent papers, however, substantiate that LA is the technique of choice in the treatment of AA in terms of clinical advantage and cost-effectiveness [1,3,5,10-15]. Notwithstanding, more than 20 years later, the benefits of LA still remain a controversial issue for many authors.

The current floundering economy of Spain (and many other European Countries) is seriously affecting health services. It is, therefore, our duty to achieve optimal efficiency in the surgical procedures we perform with the aim of doing the best for our patients at a minimal cost. Thus, the aim of our study is to present our LA technique and determine if LA should be the technique of choice in any case of AA because of its lower cost, shorter hospital stay and lower morbidity (higher cost-effectiveness), even though in principle it may seem to be a more expensive technique than OA due to the need for high cost disposable laparoscopic instruments.

## Materials and methods

We prospectively evaluated all cases of AA operated in the Department of General and Digestive System Surgery of the Marina Baixa Medical Center, in Alicante (Spain), over a 12 month period (between February 2011 and February 2012). All patients were initially evaluated by a physician of the Emergency Department and underwent laboratory blood tests (cell count, biochemistry and coagulation test); most of them underwent abdominal CAT-scan or abdominal ultrasonography in an attempt to diagnose AA. When AA was confirmed by imaging or there was otherwise strong enough cause for suspicion regardless of the result of the radiological imaging test, then subsequent consultation by the duty surgeon determined whether or not surgical invention would take place. Only two surgeons in the department suitably qualified and with vast experience in advanced laparoscopy, performed LA using the same technique in all their cases. OA was performed by the rest of the surgeons.

LA was carried out under general anesthetic. A dose of prophylactic clavulanate-amoxicillin (2 g-200 mg) was given to all cases (except allergies) and the skin was shaved 30 minutes prior to surgery. The surgical field was dabbed with iodine solution. Open laparoscopy was initiated by placing a Hasson trocar immediately below the umbilicus and a 5 mm trocar in each iliac fossa. Where any free liquid was found, a sample for bacteriological culture was obtained and the rest of it was completely aspirated. After identification of the appendix, the mesoappendix was coagulated and cut by means of monopolar cautery, particularly the appendicular artery. The appendix was ligated by means of a transfixive stitch at the base with a 2/0 absorbable suture and the specimen was then cut and extracted by using the finger of a powder-free surgical glove in order to prevent any contamination of the peritoneal cavity or the surgical wound by the infected specimen. Finally, a purse-string suture was placed on the caecum to invaginate the appendicular stump and the cavity was then gently irrigated with at least 2 liters of warm (38°C) normal saline solution and aspirated, focusing on the right iliac fossa, Douglas pouch, the right flank and perihepatic space. In case of widespread inflammation, a penrose drain was placed on the right iliac fossa according to the surgeon’s criterion. Trocars were then removed, the umbilical hole was closed by means of a 1 Ti-Cron® suture (Covidien Wound Closure) and the skin was sutured with surgical staples.

OA requires the same preparation and prophylaxis. The incision may vary depending on the surgeon’s criteria and the characteristics of the patient (Mc Burney, Rockey-Davis or right para-rectal incision). Mesoappendix was ligated by means of a 2/0 silk and a purse-string suture of the same material was placed on the caecum to invaginate the appendicular stump. Lavage with warm saline solution and surgical sponges was performed as deep as the incision would allow. Lavage of the wound with saline solution was carried out followed by skin closure by means of surgical staples.

All data regarding length of hospital stay, morbidity, need for re-consultation in the emergency department after hospital discharge and hospital re-admission were recorded. Patients were classified into four groups according to the type of AA: catarrhalis-phlegmonous appendicitis(FA), gangrenous appendicitis(GA), appendicular plastron with or without localized abscess (PA) and diffuse appendicular peritonitis (DP). Each group was divided into LA and OA subgroups. Surgical wound infection was defined when a positive culture or purulent discharge was detected or when the wound presented pain or tenderness, localized swelling, redness, or heat, and the incision was deliberately probed by the surgeon resulting in a positive wound culture.

Surgical time was measured from the moment of the skin incision until the closure of the skin.

The costs were calculated based on disposable material (Table 1) and hospital stay costs were calculated by means of the center’s clinical information program (“Discharges”), which calculates the cost for the length of stay (LOS), in accordance with the tax regulations of the Valencian regional government, regarding fees for public services based on the DRG and LOS [16].

**Table 1:** Cost of the material used in OA and LA

**OPEN APPENDECTOMY**	**Nr. UNITS**	**TOTAL**
2/0 silk suture	3	0.4 €
2/0 braided absorbable suture	2	4.3 €
Suction device	1	2.3 €
**TOTAL**		**7 €**
**LAPAROSCOPIC APPENDECTOMY**		
Hasson Trocar	1	37 €
5 mm Trocar	2	70 €
Endoclinch	1	75 €
Lap. Irrigation/suction device	1s	70 €
Biosyn® 3/0	2	4.3 €
1-Ti-Cron® suture	1	3 €
**TOTAL**		**259.3 €**
**DIFFERENTIAL**		**+ 252.3 €**

The material for LA is 252.3 Euros more expensive than for OA.

Statistical analysis was carried out by means of SPSS 9.0, calculating Student’s t to compare means and the Chi-square test for the Odds-ratio. The study was approved by the Management and Ethics Department of the Center.

## Results

One hundred and forty-nine patients underwent surgery. Six cases were excluded when the operation ruled out AA. The average age of the 142 patients was 31 years (age range 7–80), 87 were male and 55 female. The indication for surgery was established in 10 cases based on those clinics with no imaging test, and in another 14 cases, in clinics with a non-conclusive radiological imaging technique. In 118 cases, indication for surgery was supported by a positive X-ray imaging test (showing AA signs). Ninety-nine patients underwent OA and 43 LA. Both groups were homogeneous and comparable in terms of age, gender and type of appendicitis.

Global hospital stay for these 142 patients amounted to 495 days and the global cost of the stay was 223.782 Euros. The mean length of stay of the LA group was 2,6 days and that of the OA group was 3,8 days (p = 0,010). Thus, LA saves 1,2 days of hospital stay on average. Mean cost of hospital stay for the LA group was 1.081 Euros and 1.799 Euros for the OA group (p = 0,002).

Among those 142 patients, 74 had a FA of which 22 underwent LA and 52 OA; Mean hospital stay was 1,8 (±1) days in the LA subgroup and 2,6 (±1,2) days in the OA subgroup (p = 0,004). Average hospital stay cost was 1.264 Euros in the OA subgroup and 702 Euros in the LA subgroup (p = 0,002).

Forty-six patients were found to have GA: 34 underwent OA and 12 LA. Mean hospital stay was 4,3 (±2,7) for the OA group and 2,7 (±1,7) for the LA group (p = 0,015). Average hospital stay cost was 2.011 Euros for the OA group and 1.000 Euros for the LA group (p = 0,006).

Nineteen patients sustained AP; thirteen of those underwent OA and 7 LA. Mean hospital stay was 7,1 (±5,6) days for OA and 5,4 (±3,1) days for LA; differences not being statistically significant due to the small sample and wide variances. Average hospital stay cost was 3.459 Euros for OA and 2.395 Euros for LA, but the differences were not significant for the same reasons.

Only 2 patients were diagnosed with acute diffuse appendicular peritonitis and both underwent LA.

The differences in hospital stay costs between AC and AL widely exceed the cost of the disposable material needed for LA (Table 1).

Differences in operating times were also found. In this way, average time for laparoscopy was 25 minutes and 34 minutes for OA (p = 0.001).

Morbidity occurred in 22 patients (Table 2), representing an overall morbidity rate of 16%. Two of these complications occurred in the LA group (5%) and 20 cases in the OA group (20%). Thus, the risk of developing a complication after surgery for AA is four times higher when the patient is operated by means of the open procedure, regardless of the type of AA (OR = 4,126; CI 95%: 1,005 a 16,941). The two complications described in the group of LA were in the subgroup of PA as following: a low output fecal fistula (that responded to non-operative management) and a surgical wound abscess. In the OA group there were 14 cases of surgical wound infection (8 of them consulted the emergency department within 30 days of hospital discharge from the surgery ward and 4 of them required readmission; the remaining cases emerged during the immediate postoperative period), 6 intra-abdominal abscesses (one presented during the immediate postoperative period and the rest required readmission), one decompensated kidney failure and one decompensated heart failure.

**Table 2:** Morbidity rates for OA and LA classified according to the type of appendicitis

	**FLEGMONOUS (n=74)**	**GANGRENOUS (n= 46)**	**APP. PLASTRON WITH/OUT ABSCESS (n=20)**	**DIFUSSE PERITONITIS (n=2)**	**TOTAL (n=142)**
LA (n=43)	0 (0%)	0 (0%)	2 (10%)	0 (0%)	2 (4.6%)
OA (n=99)	5 (6.7%)	9 (19.6%)	6 (30%)	0 (0%)	20 (20.2%)
					22 (15.5%)

## Discussion

Appendectomy has been the treatment of choice for AA since it was described by McBurney in 1894. Semm described the laparoscopic approach for treating AA over 20 years ago [2], nevertheless, LA has not been widely accepted because many studies at the end of the 20^th^ century and the beginning of the 21^st^ century failed to prove the superiority of LA over OA for several reasons [17-20]; for example, at that time, it was found that LA required longer operating times than OA, consumed more resources in terms of disposable material (initially, endoscopic stapling devices were routinely used), hospital stay was similar and time taken to return to normal activity was not much different for either technique. All these reasons overshadowed any beneficial effect of LA on cosmetic results or wound complications. But more recently, many papers have been published with substantially different results supporting LA as the technique of choice for all cases of AA instead of OA [1,3,6-15,21]. In our study, we have analyzed the operating time and we have found differences in favor of LA. In this aspect, the latest studies do not find any differences between both types of technique regarding operating times [1,3,22,23] and some even found shorter operating times for LA [24]. Hence, some authors have highlighted a progressive drop in operating time due to the learning curve [9] and so they have attributed the longer operating times described in earlier papers to a shorter experience in laparoscopy at the outset.

One of the arguments that repeatedly supports the use of LA as opposed to OA is its shorter LOS [1,3,9,11-14,24]. In our series, LOS for LA is 1,2 days shorter than for OA on average and we also found that the higher the degree of AA , the more days of hospital stay LA saves. This is partly explained by the higher morbidity rate that marks the highest degrees of AA. Furthermore, the fact that the most complicated DRGs have a higher cost per day according to the regional government tax regulations regarding public service costs [16] and that these are marked with longer LOS makes the health costs for these groups shoot up. The same effect has been described in a multicenter study published by Hass [3]; the study pointed out that despite a higher cost of the material needed for LA, the cost of the entire procedure is still 27,6% lower than OA due to similar operating times, lower LOS and a morbidity rate 5% lower for LA. Regarding the costs of the laparoscopy material, Chu [11] stated that the use of endoscopic linear staplers is responsible for the elevated cost of LA (300$ per firing), whereas other methods for ligating the appendix and the mesoappendix are much cheaper, thus any of those more cost-effective methods ought to be used instead of endoscopic linear staplers. Thermocoagulation of the mesoappendix (by means of bipolar device of electrocautery) has been shown to be an effective and much cheaper mean to control the appendicular artery [25-28] and, indeed, we have registered no hemorrhagic complications related to this method of controlling the appendicular artery. For the appendicular stump, we have used an intracavitary “handmade” suture as described because it is safe and the cost is far lower. Some authors maintain that the stapling takes only a few seconds (much less than a handmade suture) but they do not bear in mind that preparing and correctly locating the device also takes a time that is not taken into consideration on endorsing this claim [29].

Therefore, the main advantage of LA is in terms of LOS and complications. For this reason, Tiwari [12] published a retrospective analysis of 208.314 patients undergoing several laparoscopic procedures (including emergency LA) stratified in different groups according to the severity of the disease and found a reduction in mortality rates, morbidity rates, ICU admissions, hospital readmissions in the following 30 postoperative days, lower LOS and significantly lower costs for all the laparoscopic procedures. Hence, the general conclusion of this large multicenter study is that laparoscopic approach for all these procedures is safe, efficient and cost-effective compared to open techniques. Gil Piedra [30] found that AL is far superior than OA in terms of complications arising in the most serious cases of AA (gangrenous and perforated).

Focusing on morbidity (Table 2), we found a rate of 5% (2 cases) for LA, which is similar to the rate described by other authors. Nevertheless, morbidity rate for OA is significantly higher than in other centers [1,5-10,13,14,23,24,29], although Vallribera published a complication rate in the same fashion [31]. In this way, there is a high incidence of intrabdominal abscesses in the OA group that might be explained by the impossibility of performing a correct and complete lavage of the abdominal cavity as performed in the OA group.

From a different point of view, many studies have proved the same advantages of AL, especially in the most complicated cases of AA [30,32-38], in pediatrics and the elderly [38], having also a diagnostic capability particularly useful in these cases (although this is a characteristic of laparoscopy in all cases where the diagnosis may not be completely clear). Some old studies have reported an increase in intraperitoneal abscesses for LA in pediatrics but this has been completely ruled out by more recent studies [32-38], asserting once more that AL is a safe and effective procedure.

Finally, we need to consider patient satisfaction; Vallribera [31] published a controlled randomized trial comparing LA and OA. In this study, a specific test to assess the quality of life perceived by the patients was used and, again, the results of the study found out that LA reduced LOS, morbidity rate, the need for analgesia in the immediate postoperative period, and improved the patients’ quality of life.

### Limitations of the study

This is a study that was performed in a small Hospital (260 beds facility). The two surgeons performing LA came from a larger and more "modern" facility and where recently employed in this is department of surgery were the rest of older surgeons were reluctant to the technique probably based on knowledge from oldest publications. Therefore, we decided to compare the results of both techniques that were being performed in the department and show that our results are consistent with the results of the latest publications that clearly shown the superiority of LA, but, unfortunately, due to the characteristics of the department, randomization for a les biased results was not possible.

## Conclusions

Nowadays, LA is the technique of choice in our environment, regardless of the type of AA, being performed by skilled surgeons, as it has emerged as a safe and cost-effective technique by reducing LOS and morbidity rates. The specific technique that we present, using no endoscopic linear stapler, is safe, cost-effective and feasible and contributes to the reduction of costs.

## Competing interests

All the authors declare that they have no conflict of interest.

## Authors’ contributions

DCN contributed the original idea of the manuscript wrote the text in all its sections and did the corrections. MJF contributed by performing about 50% of the laparoscopic intervention and the implementation of the material. AIR contributed by collecting all the data. All authors read and approved the final manuscript.
